# Decolonizing prisons: Indigenized programming and a critique of critical prison studies

**DOI:** 10.1177/26326663231188203

**Published:** 2023-07-31

**Authors:** Justin Everett Cobain Tetrault

**Affiliations:** 1 2200 Law, Crime and Justice Studies, University of Alberta—Augustana Campus, Camrose, Canada

**Keywords:** Decolonization, indigenization, colonialism, critical carceral studies, critical criminology, Indigenous

## Abstract

Critical prison studies (CPS) is increasingly influential in scholarly discussions about decolonizing prisons. Proponents of CPS largely oppose the idea that cultural prison programs are decolonial, which include courses teaching Indigenous cultures and colonial history, prisons facilitating spirituality, involving Elders and communities in rehabilitation, and specialized prisons called “healing lodges.” Most CPS scholars writing on the topic disparage these initiatives as assimilationist and thus a weapon of cultural genocide. For these scholars, decolonizing prisons is impossible. CPS arguments against Indigenized programming are premised on abolitionist theory rather than a discernible decolonial method centring the perspectives of Indigenous peoples affected by prisons. I explore the accuracy and utility of CPS scholars’ arguments against Indigenized programming, drawing from 587 interviews with incarcerated men and women detained inside six prisons across Western Canada. Almost 40% of these participants identified as Indigenous. Indigenous interviewees nearly universally praised Indigenized programs for how they can help heal and empower those affected by colonial violence. Participants desired more cultural programming and easier access to such resources. More research is needed on how prisons develop and implement cultural programming, but existing empirical works suggest that these initiatives, while flawed, support the dignity of incarcerated Indigenous peoples. My critique is a call for criminologists writing on Indigenous issues to consider the ambiguity and tensions of decolonial processes and to premise their arguments foremost on methods centring Indigenous peoples affected by incarceration.

## Introduction: What is “Indigenizing prisons”?

Canada has been “Indigenizing”^1^ prisons since the 1970s, which involves courses teaching Indigenous cultures and history to incarcerated people, facilitating spirituality, involving Indigenous Elders and communities in rehabilitation, and creating special Indigenized prisons called “healing lodges.” Settler colonial states such as New Zealand, Australia, and the United States have introduced similar initiatives.

In Canada, we can trace the origins of Indigenized programming to the prisoner-led Native Brotherhood movement, which began in 1958 at Stony Mountain Penitentiary in Manitoba (Adema, 2012, 2018). The Native Brotherhood—and later, Sisterhood—raised awareness of Indigenous issues and pressured prison administrators to officially recognize and sanction traditional Indigenous sweat and pipe ceremonies. The movement also encouraged healing through ceremony, cultural handicrafts, group therapy, and alcohol and drug treatment programs and used hunger and work strikes to protest harsh prison conditions (Adema, 2012, 2016). Indigenous Elders also played a key role in the movement. Perhaps most famous is the work of Elder Arthur Solomon (1993, 1994) whose essays and poems drew attention to the issues facing incarcerated Indigenous peoples (Adema, 2012). Solomon called Canada's prison system a “crime,” “abomination,” “evil empire,” and “white racist institution” and helped found the International Conference on Prison Abolition (Delisle et al., 2015). Solomon (1994) also advocated for helping incarcerated Indigenous peoples live dignified lives and worked inside federal prisons where he developed an Indigenized prison curriculum in Ontario (4; Adema, 2018: 57).

In 1975, the Brotherhood and Sisterhood advanced formal recommendations, such as cultural programming for prisoners, Indigenous rights to practicing spirituality in prison, and Indigenous employment in the criminal justice system (Adema, 2018: 252). These recommendations are the foundation of modern Indigenized prison programs in Canada. Advocates stressed that Indigenous communities or leaders have control over these initiatives, but the Indigenous influence on them is limited. For example, Elder visits in Canada began as a volunteer program, but as of 2013, the Correctional Service of Canada (CSC) hires Elders as paid employees. This development formalizes these positions and provides Elders with a salary but also makes them more constrained by institutional rules (Adema, 2018: 262; Vecchio, 2018: 105). Scholars and advocates, as well as the Truth and Reconciliation Commission (TRC, 2015) and National Inquiry (2019) into Missing and Murdered Indigenous Women and Girls (MMIWG), have called for more Indigenous control over cultural programming (see also Ewert, 2022).

Criminologists have been rightfully skeptical of whether Indigenized programming is “decolonial,” given that the modern prison is a Western-colonial invention and that incarceration rates of Indigenous peoples in Canada have increased by 43% over the last decade (Zinger, 2020). We also know remarkably little about how prisons develop and implement Indigenized programming and how incarcerated people experience these initiatives. Despite our limited knowledge, some academics confidently argue that such programs advance colonialism. Over the last decade, critical prison studies (CPS) (also called anti-carceral criminology or critical carceral studies) has come to dominate the scholarly discussion^2^ on decolonizing prisons. CPS is an umbrella term referring to interdisciplinary scholarship premised on the idea that prisons and penal or “carceral” practices are fundamentally unjust and harmful and ought to be abolished. Canadian researchers have been at the forefront of much of this commentary, with many arguing that Indigenized programming is, at best, unproductive and, at worst, genocidal.

In this article, I draw from the findings of the University of Alberta Prison Project project to detail several problems with how CPS scholars frame efforts to Indigenize prisons. I do so by drawing upon interviews with 587 incarcerated men and women detained inside six prisons across Western Canada. Our research team conducted demographic surveys at four of the six prisons, where 39% of our survey sample participants identified as Indigenous (160 people), with Indigenous women being the most severely overrepresented racialized group in our study, making up 53% of our sample population of incarcerated women (54 women). This article focuses explicitly on these Indigenous participants’ observations. In stark contrast to the prevailing CPS literature, our participants almost universally celebrated Indigenized programming for how it can contribute to the healing and empowerment of incarcerated Indigenous peoples affected by colonial violence. A limitation of this research is that our team did not investigate the specifics of the Indigenization process. My arguments are premised on our participants’ stated experiences with these programs. While more research is needed on how prisons develop and implement Indigenized resources, this study reveals the complexities, ambiguities, and tensions that come with decolonial processes affected by colonial violence (see also Tetrault 2022).

## Literature review: CPS and abolitionism are not inherently decolonial

### Indigenizing prisons and the growing popularity of CPS

Academic writings on “Indigenization” and its limits have origins in South American scholarship, such as works by Colombian scholars Friede et al. (1975). South American scholars have also critiqued certain Indigenization processes as being “state Indigenism” for imposing assimilationist identities on Indigenous peoples (Garcia-Bravo, 2009: 48). Garcia-Bravo (2009) explains how, since the 1970s, Indigenous peoples have become more involved in Indigenization processes that challenge state assimilation, which Latin American scholars refer to as “critical Indigenism”(49).

Empirical studies on Indigenous peoples’ experiences with Indigenized prison programs are rare, as prisons are a difficult research site to access and in which to conduct ethnographic work. Yuen and Pedlar (2009) provide one of the only recent qualitative studies engaging with incarcerated Indigenous women in Canada. Their findings show that Indigenized programs helped participants overcome the stigma they associated with Indigenous identity, helped them connect to or discover their culture for the first time, and helped participants heal emotionally and spiritually. Empirical work on partnerships between prisons and Indigenous communities also generated some positive findings. For instance, Timler et al.'s (2019) study of a prison-community gardening program showed that incarcerated men who were mostly Indigenous found “substantial benefits” from the experience, as participants reported lower stress, greater self-worth and self-esteem, and an improved diet. The program also helped participants to imagine a meaningful future after release (449,458; see also Varcoe et al., 2020).

Over the last decade, scholarship on incarcerated Indigenous peoples in Canada has been increasingly dominated by CPS, which tends to draw different conclusions. This approach is founded on prison abolitionism (Seigel, 2018: 136) and begins from the moral premise that prisons are absolutely and irredeemably oppressive (immoral) spaces. As Canadian penal abolitionists Carrier et al. (2018) argue, prisons are fundamentally immoral because they do not achieve their proposed outcomes (making people and society safer) yet continue to violate the dignity of the individual. As such, we must abolish prisons. Academic approaches to prison abolition are traceable to arguments made by Norwegian sociologist Thomas Mathiesen in his book *The Politics of Abolition* (1974), as well as works by Christie (1977), Hulsman (1986), and others.

Building on arguments by the preceding critical criminologists, CPS scholars purport to offer “alternative” understandings of prison and incarceration compared to dominant criminological discourse. As Brown and Schept (2017) explain, CPS is concerned with “destabilizing criminological common sense” and developing “alternative vocabularies and analyses from which to begin to work our way out of the carceral state” (441,444). These critiques often involve reimagining or challenging prison reforms, such as Indigenizing prisons.

Building on the ideas of Austrian-French scholar André Gorz (1967), abolitionists tend to analyze policy changes to prisons and punishment through the theoretical framework of “reformist” and “non-reformist” changes. For abolitionists, “reformist reforms” are policy changes that strengthen the status quo, in this case by legitimizing, improving, maintaining, or salvaging the prison system. In contrast, “non-reformist reforms” (also called revolutionary or abolitionist reforms) are policy changes that do not in and of themselves abolish prisons but help dismantle or reduce carceral practices. A subset of CPS investigates whether certain policies are “reformist” or “non-reformist,” as the distinction is often unclear.

CPS scholars widely regard Indigenizing prisons as a reformist reform. These researchers have sought to “destabilize” the idea that such initiatives are decolonial and serve the interests of Indigenous peoples. Instead, most CPS scholars argue (or heavily imply) that Indigenized programming advances cultural genocide.^3^ This CPS critique can be broken down into the following four points:

1. *Prisons “impose” Indigenous programming on prisoners.* This position relies heavily on Martel and Brassard's (2008) foundational critique of Indigenizing prisons in Canada.^4^ First, the authors argue that Canadian prisons ignore the diversity of Indigenous peoples, as programming promotes a simplified and “overgeneralized” pan-Indigenous identity. Second, Martel and Brassard (2008) claim that because sentences and release consideration sometimes require that prisoners take programming, prisons therefore “impose” a pan-Indigenous identity on incarcerated people (357). Martel and Brassard (2008) support these arguments based on 25 interviews with formerly incarcerated women, some of whom performed the pan-Indigenous identity to increase their chances of release, while others rejected it completely (357). A follow-up study by Martel et al. (2011) builds on their original critique, arguing that Indigenizing prisons promote contradictory ideas of healing and punishment and that prison-based efforts to teach about Indigenous culture and identity are foremost about risk management by the prison.
2. *Indigenized prison teachings are assimilationist/genocidal.* As one of the only critiques based on substantial interactions with Indigenous peoples, Martel and Brassard's (2008) study is widely influential for Canadian CPS scholars arguing that Indigenized programming represents a continuation of cultural genocide. Citing the preceding studies, McGuire and Murdoch (2021) argue that Indigenized prison initiatives—including prisons facilitating ceremony—are “assimilatory destructive programs” that “reinforce and perpetuate harm” (12). They describe the prison's cultural teachings about Indigenous identity as a “state-imposed” “fallacy” comparable to how Canada's Indian Act legally defined “Indians” for genocidal purposes (4–6, 8, 12). Struthers Montford and Moore (2018) assert that Canadian prisons are “spaces of ‘extreme pedagogy’ directed to colonial ends” (657) and that Indigenized programming therefore “functions to impose and manipulate identities…[to]advance and justify the colonial project” (644, 658). Nichols (2014) argues that the “cultural accommodation” of prisons is comparable to residential schools, referring to how the Canadian government forcibly removed Indigenous children from their families, imposed Euro-Canadian culture on them, demonized Indigenous cultures, and punished children who practiced their culture, including speaking Indigenous languages (448). Marques and Monchalin (2020) similarly suggest that Indigenized programming amounts to the “benevolent destruction” of Indigenous peoples (94) and that the prison's cultural teachings align with the efforts of Duncan Campbell Scott, the architect of residential schools (92). Martel et al. (2011) argue that Indigenous communities' involvement in prisons and healing lodges is comparable to “indirect rule” by the British Empire and that such initiatives can make Indigenous leaders and community members into “agency Indians” of the state (250).
3. *Canadian prisons exert an absolute form of colonial oppression and are a continuation of a genocidal project.* CPS scholars writing on this subject tend to argue or strongly imply that prisons themselves are institutions of cultural genocide. Their critiques tend to build on broader abolitionist theories of incarceration and colonization, rather than Indigenous studies literature on cultural genocide.
  CPS scholars writing on Indigenizing prisons tend to represent prisons as all-consuming institutions that systematically suppress or undermine Indigenous agency and resistance in a near-absolute way. For most CPS scholars, just as prisons are near-total in their power and oppressiveness, prisons are also totalizing in their colonialism. Rinaldi and Marques (2020), for example, conceptualize Canadian prisons as the “carceral necropolis” or “death-world,” meaning:

[Canadian prisons are a]colonized space that marks and treats populations as the living dead[…]built on the necropolitical premise of the devaluation and presumed disposability of incarcerated—and over-incarcerated Black and Indigenous—populations (36).[…][In Canadian prisons, a]person's life has been reduced to a biological fact, stripped of potentialities, denied conditions of flourishment thought wasted on them. (39)

Neely (2021) similarly argues that prisons have a “death ethic” and are “inherently eliminatory,” as they are an extension of the settler colonial state, which is the “macrological codification of a permanent war against Indigenous bodies” (74,90; see also Guenther, 2021; Marques and Monchalin, 2020: 83). Guenther (2023) argues that prisons are a “genocidal and ecocidal form of *colonial worlding*” (85, their emphasis). Blagg and Anthony (2019) argue that prisons are “designed to further the colonial project of Indigenous extinguishment” and reduce Indigenous peoples to “bare life” (160,162).

For most CPS scholars, the prison's absolute colonial power makes it indistinguishable from earlier colonial institutions, thereby positioning the prison as a site of ongoing genocide. Nichols (2014) argues that today's settler colonial state is as “coercive as ever” and has “shifted its site of operation, perhaps most symbolically from the residential school to prison”—suggesting that Canadian prisons and residential schools are analogous in their colonial power, aims, and effects (448). Marques and Monchalin (2020) argue that Canadian prisons are a “reinvented form of colonial governance … serving the state's unchanged objective of addressing, or eliminating, the so-called Indian problem” (92) (see also Bird, 2021: 111). McGuire and Murdoch (2021) and Marques and Monchalin (2020) argue that modern forms of imprisonment constitute a “carceral genocide” or a continued “extermination” of Indigenous peoples (Marques and Monchalin, 2020: 95). Struthers Montford and Moore (2018) similarly assert that Canadian prisons are “the new reserve” and “deployed” to “guard colonial sovereignty” and “prevent Indigenous sovereignty”(643,644). Rinaldi and Marques (2020) claim that prisons are a “Canadian colonial tool that extends the historical work of separating out and disappearing Indigenous populations from the white settler body politic” (40) (see also Evans, 2021; Monchalin, 2016).

4. *Prisons cannot be reformed and therefore cannot be Indigenized or decolonized; only abolition is decolonial.* Most CPS scholars do not explicitly reject policies that can improve prison conditions, but their research publications also typically do not advocate for such reforms. Abolitionists and CPS scholars widely present prison reforms such as Indigenization as a “carceral logic” distracting from the immorality of prison while reproducing the legitimacy and preservation of the institution and other “carceral practices” (Brown and Schept, 2017: 441,447; see also Evans, 2021). Piché et al. (2017), for instance, claim that Indigenized programming “neutralizes” critique of prisons “while negating the advancement of transformative alternatives” (35).
  These critiques of reform extend into broader conversations about decolonizing prisons. For nearly all CPS scholars writing on the subject, Canadian prisons—understood as inherently and exclusively colonial spaces—cannot be Indigenized or decolonized in any respect. Marques and Monchalin (2020) argue that such reforms “work within a colonialist and necropolitical framework” (93). Scott et al. (2021) argue that it is “impossible” to Indigenize and decolonize prisons (142–43). Guenther (2021) posits that modern criminal justice “reframes” assimilation as rehabilitation, concluding that the “rhetoric of protection or care” for Indigenous peoples represents little more than the “ongoing violence of conquest” (101). Some authors suggest that these programs are illegitimately Indigenous. Struthers Montford and Taylor (2021) refer to these practices as the “supposed” Indigenization of prisons (6; see also Piché et al., 2017). McGuire and Murdoch (2021) call the Indigenous identity taught in prison a “Halloween costume” (101; see also Piché et al., 2017; Struthers Montford and Moore, 2018). For most CPS scholars, expanding or improving Indigenized resources does little more than strengthen colonialism. Marques and Monchalin (2020), for instance, argue that investing in Indigenized programming reproduces colonial logic and results in “carceral expansion” (92; see also Piché et al., 2017; Struthers Montford and Moore, 2018: 640–41).

In light of such positions, most CPS scholars see the only legitimate form of decolonizing prisons as entailing abolition. Nichols (2014), for instance, argues that prison abolitionism “announce itself as decolonization” (455, see also Struthers Montford and Taylor, 2021: 2). Consequently, rather than advancing pragmatic recommendations to improve the living conditions of incarcerated Indigenous peoples, most CPS scholars advocate for or heavily imply abolishing prisons (see, e.g., Bird, 2021: 116; Blagg and Anthony, 2019: 154; Marques and Monchalin, 2020: 97; Rinaldi and Marques, 2020: 12). As Seigel (2018) puts it: CPS is “abolitionist by implication if not in outright and explicit intention” (136).

### Methodological issues with CPS’ approach to decolonization

Notably missing from CPS scholars’ critiques of cultural programming is a discernable methodology showing how they centred the opinions of Indigenous peoples affected by prisons.^6^ Indigenizing prisons warrants critique, but CPS’ assessments of these initiatives tend to entail unequivocal sweeping statements about such programs based on remarkably little original qualitative or quantitative data. Instead, prominent CPS arguments against Indigenized resources appeal foremost to the morality of preestablished political commitments, with scholars typically making the circular argument that programming is oppressive because anything related to prisons is inevitably and only oppressive. This approach is encapsulated in Marques and Monchalin's (2020) argument that while investing in Indigenized programming is “seductive,” we should oppose such measures because “the result is carceral expansion wherein more individuals become incarcerated, rather that diverted, due to the best services and supports being those behind prison walls” (92). There are many problems with such assertions. First, to support their position, the authors only cite one study by Piché et al. (2017) which shows that prison authorities discuss Indigenized resources when promoting prison construction in Canada. This fact, however, can in no way lead to the conclusion that improved programming *causes* prison expansion. There is also minimal research on Canadian prison services generally, and no criteria by which we might deem any such programs to be the “best.”

Second, Marques and Monchalin's (2020) argument is based foremost on moral reasoning rather than an empirical assessment of how programming affects Indigenous peoples. The starting point for Marques and Monchalin (2020) is that incarceration is a “tactic of control, assimilation, and extermination” (95) and, by this logic, expanding programming makes genocide more efficient. This premise leads to their circular argument that we should not invest in Indigenized resources because doing so causes prison expansion, which is inherently immoral, because prisons are immoral. Moreover, what constitutes “prison expansion” in CPS is poorly defined, especially as authors increasingly adopt the more ambiguous and normatively loaded language of “carcerality” to dismiss or condemn an increasingly large and diverse array of institutional forms and practices (see Brown and Schept, 2017).

CPS scholars tend to represent their academic approach as the central authority on decolonization, rather than decolonization being an open-ended and decentralized process engaged in and decided upon by Indigenous peoples and communities. This is evident in how nearly all CPS’ critiques of Indigenizing prisons foreground abolitionist theory over decolonial research methods. In other words, nearly all CPS scholars avoid detailing how their critiques centred the perspectives of Indigenous peoples affected by prisons nor how they weighed Indigenous peoples’ diverse opinions about responding to mass incarceration, such as discussing how CPS arguments conflict with recommendations by the TRC and National Inquiry into MMIWG. The TRC report is derived from interactions with over 70 Indigenous communities conducted over 5 years. The final report of the national inquiry is based on over 2300 recorded experiences of Indigenous peoples recorded over 2 years. Both inquiries explicitly advocate for pragmatic policy reforms to mitigate the challenges faced by incarcerated Indigenous people, including the type of therapeutic and healing efforts in prison that CPS scholars often ignore or oppose. The certainty with which some CPS scholars assert their arguments suggests there is nothing further to learn from Indigenous peoples and their experiences with prisons.

### The consequences of critical prison scholars’ critiques

CPS critiques are concerning because they have nurtured a discourse widely dismissive of, if not hostile toward Indigenized programming on questionable empirical and decolonial grounds. These critiques and dismissals of Indigenizing prisons are not benign or inconsequential but can have undesirable and counterproductive consequences. At the most basic level, the sheer number of works that CPS scholars produce on this topic means that they are profoundly shaping academic perceptions of Indigenizing prisons. For example, the piece by McGuire and Murdoch (2021) characterizing cultural programming as “destructive” and advancing “genocidal carcerality” won *“Punishment & Society's”* 2022 “best article prize” and, at the time of this writing, is the “most read” article in the journal over the last 6 months, having been viewed or downloaded over 24,100 times from the SAGE Journals website. A generation of critically inclined students interested in learning about, and hopefully improving the situation of incarcerated Indigenous peoples, will encounter a scholarly literature dominated by works portraying Indigenized resources as “destructive” but minimal empirical research supporting pragmatic programming options that many Indigenous peoples and communities have advocated for.

CPS scholars’ influence on this subject can also be more immediate and disconcerting. This is apparent in a recent article by Tubex et al. (2021), where they interviewed Indigenous peoples released from prison and living in remote communities in Australia. In that article, the authors contemplate the prospect of introducing to Australia the type of Elder-assisted parole hearings and healing lodges that exist in Canada. However, after reviewing the Canadian CPS articles cited in this section, the authors conclude that such Indigenized programming is “forced” (294), “ignores the diversity and authenticity of Aboriginality,” and “increases the power of the criminal legal system over Indigenous lives and communities” (296). They consequently do not recommend Elder-assisted parole hearings nor healing lodges in Australia, despite initially characterizing the practices as “very promising and *in line with what participants in our research were asking for*” (294, emphasis added).

Altogether, contemporary scholarship tends heavily toward arguing that efforts to Indigenize prisons are undesirable, harmful, and often genocidal. Our interviews with incarcerated Indigenous peoples complicate and challenge these claims.

## Methods and sample: Qualitative interviews with incarcerated Indigenous peoples

The data for this article come from the University of Alberta Prison Project, a multi-year study of life experiences inside Western Canadian prisons on which I am a collaborator. This article draws from semi-structured interviews our team conducted with 587 incarcerated men and women, drawing specifically on the large number of Indigenous participants in our sample. I personally conducted semi-structured interviews with over 100 incarcerated people in four provincial prisons and two federal prisons. Among our survey sample (413 people), 39% of participants identified as Indigenous (*N*  =  160), with Indigenous women being the most severely overrepresented racialized group in our study, making up 53% of our sample population of incarcerated women. We interviewed participants who experienced residential schools and whose family members had gone to these institutions (25% of our Indigenous participants). Of the Indigenous respondents, 34% lived on reserves, and 34% had experienced the foster system. Many of these people were victims of the “Sixties Scoop,” which involved the Canadian government empowering child services to remove Indigenous children from their families in order to assimilate them.

We spent 3 or 4 weeks at each institution collecting data. Our research team comprised of the two principal investigators, and six to eight graduate students made up equal numbers of men and women. Team members had varied ethnic backgrounds, and the racial composition of the research team did not appear to meaningfully affect our participation rate or the interview dynamics. I am Red River Métis and a direct descendent of Eulalie Gladu, sister to Métis leader Louis Riel.^7^, and the only Indigenous researcher on our team.

We recruited participants by making announcements on the living units of each prison. While our interview questionnaire asked generalized questions about daily prison life and experiences, we allowed participants to drive the discussion based on their interests. For example, we typically started interviews by asking “can you tell us about yourself? As much or as little as you’re comfortable sharing.” Indigenous participants often discussed their culture or community in these opening discussions, allowing us to probe about Indigenized prison resources. We asked all participants about their experiences with and opinions on prison programs and conducted demographic surveys at four of the six prisons, which asked quantitative and qualitative questions related to Indigenous identity, including questions about their family, tribe, reserve, and experiences in residential schools or foster care, if applicable.^8^

We audio-recorded all interviews with the consent of participants, which took place in private interview rooms and lasted approximately 90 min. Some interviews took multiple sessions due to interruptions or longer lengths (some exceeded 4 h). Our team transcribed all interviews verbatim and coded the transcripts using Nvivo Pro 11 and 12. This project received ethical approval from the University of Alberta Research Ethics Board. We have anonymized our participants and used pseudonyms throughout all documents.

## Analytical framework: Indigenous research methods and decolonial theory

My analysis borrows from my work on the University of Alberta Department of Sociology's TRC Calls to Action Committee (2023) since 2019, where I am the lead author of a “living document” outlining how decolonization applies to sociological pedagogy and research. I developed this document over two years with help from committee members and guidance from local Elders and Indigenous community leaders. Based on these interactions and borrowing from decolonial theory, I define “decolonization” as involving two major components: visibility and action. First, decolonial approaches must make Indigenous peoples, knowledge, and/or issues visible, which necessitates critiques of colonialism, Eurocentrism, and settler societies. Second, decolonial approaches work with Indigenous peoples to increase their sovereignty, self-determination, and material well-being, which involves centring Indigenous perspectives and helping identify or address their needs and interests.

The preceding approach to decolonization has various implications for research. First, the decolonial researcher rejects the idea of knowledge creation being politically “neutral” and foregrounds how colonialism has affected nearly all aspects of settler societies and Indigenous relations, sometimes called “coloniality” (Dimou, 2021). Second, they recognize the incredible diversity of Indigenous peoples and do not assume to know in advance the substance of their interests. This means that decolonial research is empirical and ideally takes the form of “activist scholarship,” where the researcher engages with Indigenous peoples to help identify and address those needs (Juris, 2007). In short, there is no obvious or “all-purpose” way to decolonize—decolonial action is a fluid and collaborative process decided by Indigenous peoples. Decolonization is also ongoing: there is no “post-colonial” institution, nor do we arrive at a decolonial “end product” (Sium et al., 2012). A more detailed outline of what I argue constitutes “decolonial research” can be found in in my previous work on this topic (see Tetrault, 2022).

## Findings

### What is Indigenized programming?

We know little about how Indigenized prison programs work in practice. There is minimal information concerning their structure or components, the content of Indigenized prison curriculum, the level of community involvement in Indigenization, or the degree that incarcerated people can access these resources. My assessment of how these programs work is based foremost on how our participants characterized their experiences.

Indigenized programming is structured, semi-structured, or unstructured. Structured programming involves instructors teaching incarcerated people in a classroom setting. These courses typically center on teaching the basic elements of Indigenous cultures and the history of colonialism in Canada and can involve therapeutic or self-help components that instructors connect to cultural traditions. Unstructured programming involves incarcerated people practicing cultural traditions alone or with other prisoners. Many of the prison units we studied, for instance, allowed incarcerated people access to sage, sweetgrass, and drums, which prisoners could use for prayer or group ceremonies. An example of semi-structured programming is Elder visits. CSC employs Elders to provide guidance to incarcerated men and women.

Finally, the most substantive form of Indigenized programming available to some of our participants was the “Pathways” program. Incarcerated people in Pathways spend multiple months in a special living unit where they have more regular access to all forms of Indigenized programming. A considerable volume of Indigenized programming is essential to incarcerated people's right to religious or spiritual accommodation. However, as I will show, access to all Indigenized resources can be limited.

### How Indigenized programming helps people connect with their culture

Indigenous participants frequently praised the different aspects of Indigenized programming for teaching them Indigenous and colonial history and helping them connect to their culture, often for the first time. Irene's biography, for instance, was common among the women in our sample. Irene experienced sexual abuse as a child. Both of her parents went to residential schools. Irene spent most of her life in group homes, treatment centers, or prison. She explains how Indigenized teachings inside a federal prison helped her connect with her Indigenous identity and to contextualize her personal struggles as stemming from colonialism:

I have been incarcerated each year since [I was 19]. I wasn’t aware of much of my history, my Aboriginal social history. I’ve only learned about all that this past year through the new incorporated programs here.

I am Native, I am Cree, I am from Alberta. I have participated in almost all the programs here[…]I’ve come from a really, high risk life style, violence, you name it – I’ve got nothing but guns and violence on my record. I completely turned my life around, I’m now in a traditional Pathways [Indigenized] unit here living a traditional life [following cultural teachings]so…I basically learned about my history and understood about colonization and residential schools and the impact thereof. I just needed to really understand how much [it impacted me] as a Native American and um, I just don’t wanna be defined by [my past]. So I made the choice to change my ways and sober up and leave all that behind and things have been really good for me.

Irene's statements are representative of the broader sample, many of whom pointed to their “lifestyle” as an important reason for avoiding aspects of their culture—lifestyles typically related to conditions of poverty and marginalization. For instance, many participants discussed how growing up around drugs or violence or being homeless or gang-involved prevented them from engaging with cultural traditions. Some participants recounted how they did not experience their culture due to being raised in the foster system. Olivia outlines her experience:

Olivia: I didn’t know nothing about spirituality when I first came here, and now, like I enjoy it. I wasn’t raised in it [my culture] because I went from [foster] home to [foster] home, you know? And then living life on the streets they don’t teach that at all. Like nobody's gonna be smudging while they're getting high [laughs]. That's just sacrilegious[…]

[Doing Indigenous programming] means working with an Elder and being open to the Elder's teachings, like from their own perspective and stuff, and[…]you do a healing plan[…]and smudging and being open to spirituality, praying and stuff. When I got out [of prison last time,] I actually got my own smudge kit and everything. Yeah, I was *that* much into it.

Interviewer: And you never had [a smudging kit] before?

Olivia: No, cuz well… I've never been sober this long before.

Olivia and others affected by child services or the “Scoops” explained how some foster or adoptive parents shamed them for being “Indian.” Murray was a provincially sentenced prisoner from a local reserve who outlined his experience with the foster system:

I used to dance, pow wow, and used to be really spiritual. I had long hair too, [but] I cut it because I was in a foster home. She [foster mother] shaved my head. That kinda was it for me from there [I stopped following my culture]. Like, that kinda brought my spirit down, I just went another path from what I was doin’.[…] here [in prison] I like to go to the sweat lodge and pray, it's fun[…]and I’m getting into [Indigenous programs] that are gonna help me.

Program teachings allowed participants to place their own personal struggles into a larger historical and colonial context, helping them to overcome feelings of stigma pertaining to their Indigenous identity and heritage.

Indigenous participants almost universally praised Indigenized resources for empowering them personally and collectively. Participants explained how doing ceremony, drumming, and making crafts (such as beadwork and painting) allowed them to express their Indigenous identity. Some participants even contrasted Indigenized resources against residential schools. Chester, for example, was a 51-year-old Cree man and residential school survivor. He explained how accessing the sweetgrass provided in prison helped him manage his childhood trauma related to the schools:

Interviewer: What was [attending residential schools] like for you?

Chester: Oh, terrible[…]I took off [ran away],three or four times, I was brought back. But when I did take off[…]I went to my Elders and I learnt Cree. My band is very tough, Cree, the protocols and the prayers in Cree. That's my strength and I’ve been consistent with my language…but I still struggle through that [trauma], you know. The abuse you go through, some of the things that happened [at the school], *oh man* (emphasis). […]

The sweetgrass here helps a lot for that, being in residential school, it helps me relax. You know, to have that.[…]I wish we had sweet grass [right now]…you know what I mean? Because…[my past] kind of overwhelms me sometimes.[…]but I’m looking forward to the pipe ceremony next Wednesday.

Altogether, Indigenous participants repeatedly informed us that Indigenized resources helped them connect to their culture and heritage and overcome stigma by helping them express themselves and feel empowered and proud of their identity.

### Trauma, learning, therapy, and support networks

Indigenized programming often has a therapeutic or healing component that aims to help incarcerated Indigenous peoples work through their traumas. Such efforts are particularly important as these individuals are more likely to be victims of severe sexual and physical violence compared to both the general public and other incarcerated populations (see Bucerius et al., 2021). While non-Indigenized programs also have therapeutic aspects, Indigenized programming contextualizes participants’ traumas or criminalized behaviors in relation to Canada's colonial policies. CSC and other government institutions refer to these teachings as “Aboriginal social history” (ASH).

 Our findings challenge the popular claim by some academics that rehabilitation is foremost coercive. None of our participants expressed this sentiment. At worst, participants sometimes complained about how the mandatory course-based programs of their “correctional plan” were unhelpful or boring. Participants never raised these claims in the context of Indigenized programming, and those who complained about programming tended to have a higher education or income.

Most participants explained that they *desire* programming that might support their mental or physical health. For example, the great majority of our participants struggled with trauma from being victims of sexual and physical violence, and many responded to such experiences by using alcohol or drugs. Indigenous participants experienced victimization and struggled with substance use at higher rates and many viewed rehabilitation for drugs or alcohol as essential to their healing. Irene, for instance, explained how Indigenized programming helped contextualize drug use as a response to unresolved traumas connected to colonialism:

And working on programs…[they] go back to where everything started and explain how these cycles of violence or cycles of addiction or cycles of oppression – whatever it is that we were affected by – and go from there, and install those alternatives that are going to give us a way to live healthy and not, you know, ways to cover up your problems[…]I know people need medicine [prescription drugs] and other stuff[illicit drugs] for their mentality[coping with trauma], but there's other ways that you can [help that]…

I think traditional ways[following Indigenous teachings] is something that helps girls especially […]So more cultural programs and more access to that is something that would benefit and take girls’ minds off using that kind of alternative[illicit and prescription drugs,]big time.

Indigenous peoples tended to be the most marginalized among our sample in relation to victimization rates, poverty and homelessness, problems with substance use, and mental and physical health struggles, among other factors. Participants who struggled the most in these areas tended to find the most value in the rehabilitative aspects of Indigenized resources. For example, Jamie was a 20-year-old Indigenous woman who was sexually abused in a group home. She ran away from the home at 14 years old and experienced homelessness ever since. In Jamie's words: “Programming? Right now I’m thinking they’re all gonna be useful. I literally haven’t learned [been taught] anything in life. I done it all on my own.” For many participants, having external support and guidance was important for developing self-respect and for healing. Dayna was in the foster system until she was 17. Her mother was a victim of the Sixties Scoop and her grandparents attended residential schools. She lived in a Pathways unit for several months and explained how the program helped foster a supportive environment:

Jail is the loneliest place and the toughest of the toughest people break.[…]I used to be house rep [at Pathways], but I’ve gone through a lot this year. I’ve lost six people over the year. And my friend was just killed. So I can’t take too many responsibilities on right now[…]I’m the drumkeeper. And I’m firekeeper, so I prepare everything for the Elder, and that's a lot of work[…][In prison] you have a bunch of damaged women, “broken dolls” is what we call them [chuckles]… and [on Pathways] we uplift the other women we live with. It's a beautiful experience[…]and we have a sharing circle every week – every Tuesday we have a sharing circle. And we, all of us women sit with the Elder and talk about what we’re going through.

As Dayna outlined, incarcerated people typically welcome Indigenized programming as it can foster a culturally attuned support network, such as having peers, program instructors, and Elders encouraging them to succeed.

### Indigenized programming access issues and needs

The incarcerated men and women we interviewed almost never criticized Indigenized resources, including the structured classroom content. Instead, respondents’ concerns centered almost exclusively on troubles accessing ceremonial materials, Indigenized courses, and Elders. Participants expressed frustration at how security protocols such as prisoners’ security status, unit lockdowns (which suspend programming), and bureaucratic barriers (such as paperwork) prevented them from using basic cultural and religious resources, sometimes for weeks or months at a time. Participants struggling the most with their colonial traumas—such as residential school survivors—tended to depend more on Indigenized resources to manage their mental and spiritual health and found security restrictions particularly egregious. When asked what might help them inside prison, Indigenous participants often called for expanded Indigenized programming and easier access to such resources. Irene's opinions on Indigenized courses reflect the broader orientation of our participants:

I think you need more programs that are run by the Elders, and that are cultural-based that have ceremonies. Like, “letting go” ceremonies, because you have so much that you hold inside and when you’re able to let it go[express yourself], you’re not just letting go the things that you talked about – you’re also letting go the things that are attached to it [traumatic histories][…] you’re able to unload everything spiritually, [and] that goes a long way too. They need more programs that are like that.

Participants also wanted staff to be better educated on Indigenous cultures and colonization, as staff typically control access to ceremonial materials and supervise cultural practices such as drumming and smudging. Rory was a remanded prisoner and explained how staff racism sometimes prevents prisoners from using Indigenized resources:

Sometimes we run out of sweet grass, we run out of our BBQ lighter, you know?[And we need more, but] they[correctional officers]don’t care. And that, too, is a big problem here, is when we want to go out and smudge a lot of the guards won’t let us.[…]some of them look down on us like: “fuck you guys, you don’t need to do that shit.”

Julian, a 35-year-old remanded Indigenous man, attributed these prejudices to a lack of basic knowledge of Indigenous culture and Canadian history:

They need[more cultural]sensitivity training. Like, the guards need to know how us Aboriginals have been through a lot of shit. And they need to actually learn some fuckin history too[…]They have no idea what [Treaty Six] is, they have no idea what the treaties are, or anything like that.

## Discussion: A decolonial critique of CPS

Our findings directly challenge the four core CPS arguments about Indigenizing prisons:

*Challenge #1: “Prisons ‘impose’ Indigenized programming on prisoners.”* CPS scholars are correct in their assertion that Indigenized programming has an inherently coercive dimension. Whether incarcerated people voluntarily use Indigenized resources, or whether participating in such programs is a condition of their release, prisoners do not “freely choose” to participate in Indigenized programming. However, asserting that prisons “impose” Indigenized teachings or identity on incarcerated men and women is too simplistic. First, this argument seems to rely on the assumption that incarcerated men and women experience Indigenized programming foremost or exclusively in top-down classroom environments. Our findings suggest that most incarcerated people struggle to access prison courses. Instead, participants explained how their experiences with Indigenized resources and connecting to their identity was a fluid and collective process that combined official programming with support networks between staff, Elders, and fellow prisoners. Moreover, scholars using the word “imposed” imply that Indigenized resources are unwelcome and forced onto resistant people by a hostile prison administration. Our participants appreciated programming that facilitated learning about Indigenous cultures and colonial history. Rather than being unwanted, imposed, or burdensome, nearly all Indigenous participants praised Indigenized programming and wanted to see these resources expanded. In short, Indigenized teachings manifest in prison in a multitude of forms and no participants framed these dynamics as “coercive.”

*Challenge #2: “Indigenized prison teachings are assimilationist/genocidal.”* CPS scholars frequently argue that Indigenized teachings in prison represent ongoing assimilation, with some comparing these initiatives to the residential school system. These arguments suggest that Indigenous people who develop or run cultural programming—including Elders—are complicit in cultural genocide. While prisons are colonial institutions, scholars cannot meaningfully equate Indigenized prison programming with the colonial oppression practiced in residential schools. Our participants occasionally explicitly contrasted the empowerment they felt as a result of Indigenized resources *against* the cultural erasure practiced in the residential school system. Indigenized teachings helped participants—especially those removed or “scooped” from their communities or families—meaningfully connect with their Indigenous culture or heritage for the first time. For other incarcerated men and women, Indigenized resources helped them overcome the stigma they felt about their Indigenous identity and feel proud about their culture and heritage. Participants also explained how the programming they received from professional instructors, peer counselors, and prison-affiliated Elders *explicitly* sought to position their marginalization and personal traumatic experiences in a wider historical and colonial context. This situation challenges any easy dismissal of such programming as being exclusively assimilationist.

Many CPS scholars have also critiqued prison programming for fostering a standardized pan-Indigenous identity, which many represent as inauthentic. This raises delicate questions about who is empowered or authorized to decide on or speak to what might “count” as authentically Indigenous. In the case of identity fostered in Canadian prisons, Indigenous leaders themselves strategically championed this pan-Indigenous approach. So, when scholars such as McGuire and Murdoch (2021) critique the pan-Indigenous identity for ignoring “the reality[…]that most unifying factors among Indigenous people are their experiences with racism, genocide, and trauma” (12), they overlook the fact that this is *precisely* why programs embrace a pan-Indigenous approach. As Adema (2012) explains, it was efforts by the Indigenous Brotherhood and Sisterhood to organize around commonalities and shared traumas that fostered this “pan-American” or “ethnic” Indigenous identity to unify and mobilize diverse incarcerated Indigenous peoples (Adema, 2012: 43–44). And while prisons can undoubtedly improve Indigenized prison programming to better accommodate cultural differences, there are more than 630 Indigenous communities representing more than 50 nations and over 70 Indigenous languages in Canada (Government of Canada, 2017), making it pragmatically impossible for every prison to offer tailored services to each unique Indigenous culture. It is also the case that many incarcerated Indigenous peoples—especially victims of the Scoops—do not know their precise ethnic or tribal background and consequently find it easy and appealing to identify with a pan-Indigenous identity reflecting Indigenous peoples’ broader historical and cultural significance and their relationship to Canadian colonization.

*Challenge #3: “Canadian prisons exert an absolute form of colonial oppression and are a continuation of a genocidal project.”* CPS scholars correctly identify the colonial lineage of the prison (Chartrand, 2019). However, what transpires in prison is not absolutely and exclusively colonial: where there are Indigenous peoples, there is agency, meaning-making, and resistance. Arguments or insinuations that prisons are exclusively and totally colonial risks undermining Indigenous peoples’ agency, self-determination, accomplishments, and resistance inside and outside the prison. CPS scholars tend to present incarcerated Indigenous peoples as perpetual victims of the prison's colonial oppression, with little, if any—room for personal growth, healing, meaning-making, and resistance—a caricature unsupported by history and a view that our participants widely rejected. As Adema (2012, 2018) and others have illustrated, Indigenous peoples, communities, and prisoners have fought for over half a century to have their cultures recognized and accommodated in Canada's prison system. Academics should not reduce these struggles and achievements to the vision of total powerlessness implied by talk of “social death,” “death ethic,” “erasure,” “disappearance,” or “necropolitics.” Contrary to the arguments of some CPS scholars, incarcerated Indigenous peoples are not “eliminated,” “dead,” “disappeared,” or “disposed of.” Conflating Indigenized programming with cultural genocide diminishes the immense labor and struggles of Indigenous peoples who fought for these initiatives and those who have been helped to heal through such resources.

To represent prisons as absolutely colonial implies that connecting to one's Indigeneity through prison programming is impossible or colonial—as though people who respond to and embrace cultural programming are suffering from false consciousness or are passive colonized subjects duped or “manipulated” into accepting an illegitimate, inauthentic, or “fallacious” Indigenous identity. Incarcerated people, however, are not “docile bodies”—they have preferences and make choices, resisting some elements of programming while embracing others.

*Challenge #4: “Prisons cannot be reformed and therefore cannot be Indigenized or decolonized. Only abolition is decolonial.”* While some CPS scholars, such as Murdocca (2020), acknowledge the complicated and nuanced work of decolonization in the criminal justice system (36), the majority of CPS scholars portray the prison as a uniquely and absolutely irredeemable colonial institution that by its very essence cannot be decolonized. This approach to understanding prisons encourages absolutist and essentialist thinking and language that needlessly circumscribes decolonial efforts and disparages Indigenous peoples' historical and ongoing struggles for reform. Such an approach reduces colonialism and decolonization of prisons to a totalizing moral dichotomy by representing prisons as absolutely colonial and abolitionism as absolutely decolonial (if not synonymous with decolonization).

As previously discussed, CPS scholars widely view Indigenized prison initiatives as insincere or falsely humanist, if not a cunningly insidious way of advancing or “improving” colonialism itself. For most CPS scholars, culturally attuned rehabilitative programs are therefore little more than a way to legitimate the prison system. While scholars such as Carrier and Piché (2015) suggest that “abolitionists certainly do not oppose ‘pragmatic’ strategies to improve prison conditions” (5), in practice, CPS scholars’ disapproval of reform is evident in the lack of pragmatic policy recommendations related to Indigenized programming. This situation is perhaps not surprising, as which CPS scholars would advocate for better Indigenized programming when most commentators on this topic essentialize the prison as servicing assimilation or genocide?

While many of Canada's CPS scholars cite the TRC report and the National Inquiry into MMIWG, their abolitionist critiques of Indigenized resources almost never discuss how we might implement the Commission's “calls to action” nor the Inquiry's “calls for justice.” Rather than using their research and publications to explore how policy changes can be used toward decolonial or “non-reformist” aims, most CPS scholars present the decolonial prescription as obvious, absolute, and pre-determined: abolish prisons. Put another way, instead of putting their critiques in conversation with policy reforms advocated for by Indigenous communities and organizations, CPS scholars tend to conclude their analyses with vague and occasionally grandiose rhetorical gestures toward abolition. Such a stance allows CPS scholars to personally absolve themselves of being complicit in what they portray as immoral criminal justice discourse and practice. In the process, their publications evade the necessary but inevitably difficult, uncomfortable, and ethically nuanced discussions about how prison programming and liberal policy can be altered in the present to support some incarcerated Indigenous peoples and help alleviate some forms of suffering.

## Conclusion: Toward decolonial prison research and “critical Indigenism”

As a Métis person whose family has been impacted by the Sixties Scoop, I am attuned to the inequities and suffering experienced by Indigenous peoples brought on by colonialism. I also do not believe that Indigenized programming represents any kind of panacea. The Canadian government has failed to reduce the incarceration rates of Indigenous peoples nor meaningfully transition power to Indigenous communities in justice and healing processes (see Zinger, 2020). We also know little about the Indigenization process concerning the creation and implementation of cultural prison programming. Nonetheless, existing work centring Indigenous peoples shows that while Indigenized programming is contradictory and flawed, pertinent Indigenous peoples find them invaluable for healing and empowerment, making these programs an Indigenous rights issue and a decolonial effort worth pursuing.

CPS scholars offer important analyses of language and sociohistorical critiques of the prison as a colonial institution, and many abolitionists engage in decolonial activism that includes advocating for prison reforms. CPS scholars are also not monolithic. I doubt the authors cited here (nor abolitionists generally) accept all the critiques directed at Indigenizing prisons. However, academic arguments representing Indigenized programming as harmful are largely uncontested in CPS, as though there is no ambiguity concerning what decolonizing prisons entails, a position which suggests that Indigenous peoples’ needs are obvious and monolithic (Poppi and Sandberg, 2023). While CPS discourse portrays Indigenized programming as an essentially harmful initiative with little or no redeeming value, our findings—as well as those of the TRC and National Inquiry—suggest that this is not the case and that the situation is far more complicated than such scholars appear willing to acknowledge.

My critique of CPS is not a rejection of prison abolitionism. Advocates such as Elder Arthur Solomon have shown that reform and abolitionism can be compatible and Indigenous abolitionists played a key role in developing the Native Brotherhood and Sisterhood movement that inspired Indigenized prison programming reforms in Canada. These actors recognized the importance of working in the prison system to ensure that currently incarcerated people can access cultural resources that give them a sense of self-worth and dignity (Adema, 2018: 51–52; Solomon, 1994). My critique concerns the tendency for CPS scholars to present strong or sweeping condemnations of Indigenizing prisons without showing how their approach considered the varied perspectives of Indigenous peoples or communities impacted by such programs. Put another way, these CPS scholars do not have a discernable decolonial methodology. Consequently, CPS scholars cannot justify why Indigenous peoples, academics, or practitioners ought to privilege CPS critiques of Indigenizing prisons over the reforms suggested by the TRC and the National Inquiry into MMIWG or by qualitative researchers who centred Indigenous peoples and communities to arrive at their conclusions.

Prisons are deeply colonial institutions, but they are not impervious to decolonial efforts. Most CPS scholars disagree, arguing or suggesting that prisons represent “pure” colonialism, while abolitionism represents “pure” decolonization, as though colonialism and decolonization are a dichotomy. They are not. As Wolfe (2006) famously argued, colonialism is not an event, but a structure of settler societies. Consequently, decolonial action *always* happens within colonial spaces and discourses, as no person or politics exists outside of colonial history and structures. Moreover, so long as Indigenous peoples exist and resist, colonial power is never absolute or pure. Like settler societies, prisons are colonial, but can have decolonial elements, however partial or flawed they might be. Instead of wholly rejecting the idea of “Indigenizing prisons,” we can work toward what South American scholars refer to as “critical Indigenism” within carceral spaces—initiatives that seek to maximize Indigenous control of institutions and challenge coloniality (Garcia-Bravo, 2009: 49).

The Native Brotherhood and Sisterhood never intended cultural programming to be a “solution” to mass incarceration. We cannot stop at Indigenizing prisons. Decolonization is an ongoing process where we identify and address tensions between colonial and decolonial knowledges, systems, and practices. Canada's justice system emphasizes retribution and domination, which inherently undermines Indigenized programming's focus on healing and empowerment. Indigenized prison programs support the dignity of incarcerated Indigenous peoples, but realizing the potential of these initiatives requires transitioning into Indigenous-led justice and healing processes beyond prisons. Decolonial prison research recognizes the diverse Indigenous perspectives on responding to mass incarceration and resists conclusive statements about Indigenous peoples’ identities, interests, or needs in that regard. My critique is a call for criminologists to consider the ambiguity and tension of decolonial processes and to premise their arguments foremost on clear decolonial methods involving meaningful engagement with Indigenous peoples affected by incarceration.

## Notes

1. “Indigenous” refers to peoples who lived on a land prior to colonization. In Canada, section 35 of the constitution recognizes three groups of Indigenous peoples: “Indians” (First Nations), Inuit, and Métis. “Indigeneity” refers to the aspects of a person or group's identity that links them to Indigenous culture(s) or what makes them meaningfully Indigenous. However, there are ongoing debates around what constitutes Indigeneity among Indigenous peoples, scholars, and governments (see Goyes and South, 2021: 119–122). For example, some Indigenous groups in Canada assert that Indigeneity requires biological heritage, while others reject this idea.

2. By “scholarly discussion” I am referring to debates and critiques of this phenomenon in academically rigorous discourse such as peer-reviewed journals. In other words, I do not consider institutional or government reports (which are sometimes written by academics) to be “scholarly discussion” in this essay.

3. Lemkin coined the term “genocide” in 1944 and argued that it involved the intentional cultural destruction of an identifiable group, which does not always involve direct mass killing. Building on Lemkin, “cultural genocide” is typically defined as the “systematic destruction of traditions, values, language, and other elements that make one group of people distinct from another” (Novic, 2016). Indigenous peoples and scholars widely agree that Canada's colonial policies constitute a cultural genocide (see TRC, 2015: 48).

4. Martel and Brassard (2008) and Martel et al. (2011) do not reject Indigenizing prisons. Their studies rightfully identify “potentially oppressive” elements of these initiatives (Martel and Brassed, 2008: 357). Indigenized programs have also changed since 2008.

6. Except for Martel and Brassard’s (2008) critique. Martel et al.'s (2011) study includes secondary interviews with “aboriginal program officers” borrowed from a different study, and the authors do not detail the data collection process.

7. Social workers abducted my father, aunts, and uncles from their family farm during the Sixties Scoop, separating them in foster homes across the country. Some of my family members have been incarcerated, and I have lost others to suicide and substance abuse traceable to Canada's colonial policies.

8. In the Findings section, I use several statements from the same participant whose opinions and personal history are representative of the broader sample. For instance, I rely on multiple excerpts from Irene's interview because she had more experience with cultural programming and working through her traumas. Those experiences helped Irene articulate many of the concerns expressed by others in a clear and concise manner (see also Tetrault, 2022 for more on the findings).
